# Bioethanol Production by Enzymatic Hydrolysis from Different Lignocellulosic Sources

**DOI:** 10.3390/molecules26030753

**Published:** 2021-02-01

**Authors:** Katja Vasić, Željko Knez, Maja Leitgeb

**Affiliations:** 1 Faculty of Chemistry and Chemical Engineering, University of Maribor, Smetanova 17, SI-2000 Maribor, Slovenia; katja.vasic@um.si (K.V.); zeljko.knez@um.si (Ž.K.); 2 Faculty of Medicine, University of Maribor, Taborska Ulica 8, SI-2000 Maribor, Slovenia

**Keywords:** enzymatic hydrolysis, bioethanol production, biofuels, lignocellulosic biomass, agricultural waste, wood feedstock, marine algae

## Abstract

As the need for non-renewable sources such as fossil fuels has increased during the last few decades, the search for sustainable and renewable alternative sources has gained growing interest. Enzymatic hydrolysis in bioethanol production presents an important step, where sugars that are fermented are obtained in the final fermentation process. In the process of enzymatic hydrolysis, more and more new effective enzymes are being researched to ensure a more cost-effective process. There are many different enzyme strategies implemented in hydrolysis protocols, where different lignocellulosic biomass, such as wood feedstocks, different agricultural wastes, and marine algae are being used as substrates for an efficient bioethanol production. This review investigates the very recent enzymatic hydrolysis pathways in bioethanol production from lignocellulosic biomass.

## 1. Introduction

The over-exploitation of our planet’s resources has worsened our environment, which is nowadays suffering from climate change more than ever. Elevated gas emissions, the greenhouse effect, and global warming have all contributed to the search for renewable sources, which are in harmony with world’s energy needs. Lignocellulosic biomass is a sustainable alternative that produces new-generation bio-based chemicals, such as biofuels, food additives, enzymes, and others [1,2,3]. Lignocellulosic biomass includes all kinds of agricultural wastes, forestry residues, and feedstocks, as well as marine algae, and it can be provided on a large-scale platform from all kinds of materials [4,5]. In general, lignocellulosic biomasses consist of lignin, cellulose, and hemicelluloses, some organic extracts and inorganic components, which are turned into ash after combustion. All those components make lignocellulosic biomass a complex group of polymers that are naturally recalcitrant to enzymatic conversion. Lignocellulosic biomass materials are constituted of renewable substrates used for bioethanol production, where such materials play a role in contributing to environmental sustainability [6]. Lignocellulosic biomass consists mostly of polymer sugars (celluloses and hemicelluloses) and lignin [7,8]. It can be broken down into simple sugars by enzymatic hydrolysis or chemically by sulfuric or other acids [9].

Due to the process that requires less energy in mild conditions, enzymatic hydrolysis is becoming a more suitable pathway in biomass hydrolysis [10]. It is an important step in converting cellulose to glucose in pretreated biomass, which is carried out by cellulose enzymes in temperature range from 40 to 50 °C, with a pH range from 4 to 5 [11]. The degree of pretreated biomass, such as lignin removal, enzyme loading, and duration of hydrolysis is highly dependent on the enzymatic hydrolysis efficiency, since the process is also highly affected by cellulose crystalline structure [12].

The enhancement of the enzymatic hydrolysis process is possible by adding non-ionic surfactants, which can change the surface properties of cellulose, as well as reduce enzyme loading. Such non-ionic surfactant is found to be polyethylene glycol (PEG), which can reportedly increase the convertability of lignocellulosic biomass for more than 30% [11,13,14]. Biofuels based on biomass have many advantages over fossil fuels: besides contributing to fuel diversity, different biofuels are accessible by different common biomass sources, have an environmentally friendly impact and potential, and provide many benefits in terms of economy and environment for all users of biofuels. Such biofuels are biodegradable and immensely contribute to sustainability. In addition, biofuels add value to migrating greenhouse gas (GHG) emissions, which provide a cleaner and more sustainable energy source with reduced air pollution. By using biomass feedstocks for bioethanol production, such actions of biomass usage enable the emerging development of rural areas in different countries, as well as increase of agricultural income. Such developing countries have more available land with favorable climate conditions and therefore minimum or at least lower labor costs. Another advantage with large-scale biofuel production for developing countries is the reduction of its oil import dependence, which contributes to international competitiveness. When referring to the production of “good” bioethanol (bioethanol being the most commonly used biofuel for transportation worldwide) in terms of reducing the GHG emissions, it is important to replace polluting fossil fuels with more environmentally friendly lignocellulosic biomass. To ensure beneficial properties of biofuels, all kinds of by-products in the production process should be properly and efficiently utilized in order to minimize the GHG effect, as well as maximize their energy. In addition, emissions (such as carbon dioxide and nitrous oxide) should be kept to a minimum in terms of pollution and fertilizers, respectively. Moreover, biofuels such as bioethanol can help reduce the carbon dioxide escalation by replacing the fossil fuels and recycling the carbon dioxide being released when combusted as fuel [15]. However, in the ever-growing biofuel industry, sustainable energy systems and energy efficiency have an important decisive part, especially when renewable energy potentials compete with high energy demands. Many different sustainability assessments have been performed over the years, describing various schemes, such as emergy, exergy, techno-economic analysis, energy accounting, and life cycle assessments (LCA). Such schemes are being employed in all biofuel production and consumption systems [16]. As fossil fuels are massive energy sources around the globe, increasing the atmospheric concentration of GHG is still a threat contributed by fossil fuels, which result in global warming and climate change. Many renewable energy policies have been in progress to reduce the carbon-intensive energy carriers. Future low-carbon strategies support different renewable energy resources; many also use industrial waste heat. As many barriers exist for the incorporation of industrial waste heat into district heating systems, Renewable Energy Directive 2018/2001/EU (RED-II) was established to address such issues, which suggest the simplification of market entries and accesses to district heating networks for third parties [17,18]. The advanced exergy analysis-based methods are most promising for the development of sustainable biofuel systems, especially its extensions, such as exergoeconomic and exergoenvironmental approaches, which provide more details about economic, environmental, and technical features of energy conversion systems. On the other hand, the emergy concept quantifies the energy available previously in direct and indirect forms. More details about such analysis are presented in an opinion paper by Tabatabaei et al. [16].

**Figure 1:**
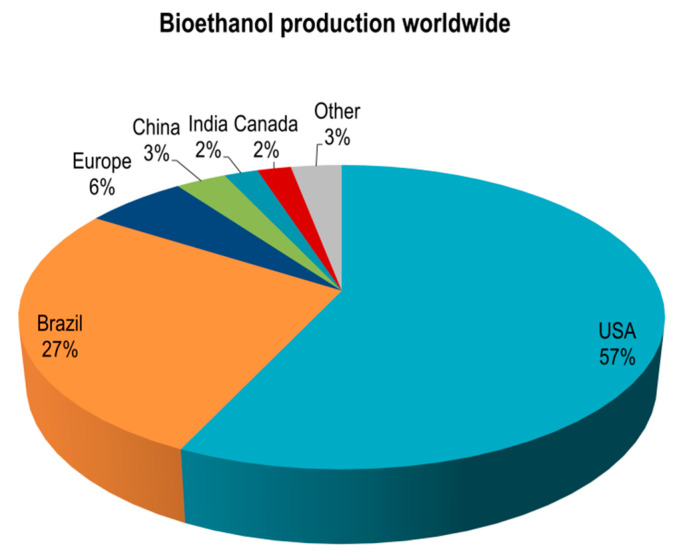
Worldwide bioethanol production.

As a result of the potential that biomass is offering, many technologies are developing toward biomass conversion into biofuels, which have the great advantages of lowering carbon emissions as well as oil dependency due to its production from renewable and organic sources [19]. As seen from Figure 1, the USA produces more than 50% of all bioethanol, while Europe’s share represents only 6%; also, each country’s share is less than 5%, while Brazil is the second largest bioethanol-producing country.

Bioethanol is the most commonly used biofuel, which is an alternative to fossil fuel and is mainly produced by the hydrolysis of cellulose from lignocellulosic biomass and by the fermentation of sugars of different lignocellulosic sources. The biodegradability and reduced toxicity of bioethanol, for which biomass is used as a primary substrate as well, are its main advantages over fossil fuels [11,15].

**Figure 2:**
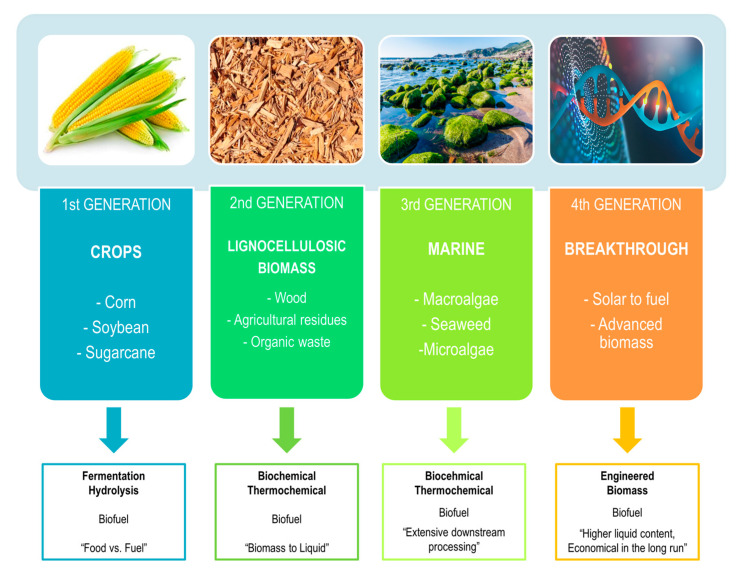
Generations of biofuels.

Advanced criteria divide the liquid biofuels based on four generation biofuels, depending on the feedstock material being used and utilized for its production (Figure 2). The primary source used for first-generation biofuels is mostly food crops, which were prepared from alcohol. The crop feedstocks used were starchy sourced materials, such as sugar cane and others. However, such feedstock presented some obstacles in the form of high market prices, since they require more chemical fertilizers to increase the biofuel yield. Second-generation biofuels are based on non-edible lignocellulosic biomasses, including whole parts of plants, such as leaves, steam, or bark, but they include also wood chips, different grasses, saw dust, paper pulp, organic wastes, and different forestry and agricultural residues. Polymeric substances, as well as cellulosic substances, are advanced sugar molecules that are found in lots of plants. Grain alcohol is obtained from these substances and is a by-product that can be used as a biofuel. The technology used for the production of second-generation-based biofuels was designed and adjusted to overcome limitations that occurred in first-generation biofuels, since they were also used and utilized as food supplements—meaning, decreasing the production of grain-based alcohol and maximizing the amount of biofuels so they can rival the competitive prices of fossil fuels. In comparison, more gas emissions are saved with lignocellulosic starch alcohol than in first-generation fuels. Considering the existing problem with land biomass feedstocks for the production of biofuels, third-generation biofuel production finds its resources in marine biomass. Such biomass requires much less land area and is good at decreasing the greenhouse emissions into the environment. For such purposes, algal biomass cultivation and farming has increased, since they give additional resources for demanding biofuel production. Improvements in the metabolic production of such biofuels enable the removal of non-fuel components as well as decrease the production costs. However, fourth-generation biofuels are the result of research and development in the fields of biotechnology, biochemistry, plant biology, geosynthesis, and its applications in metabolic and genetic engineering, as they try to cover carbon capture and storage techniques by developing advanced methods for the production of biofuels. Therefore, different bio or genetically engineered biomass feedstocks, such as algae, trees, and plants are developed that are capable of storing and managing carbon release. Thermal energy and power is being utilized by sustainable resources in form of wind, solar, geothermal and hydro energy [20,21,22]. Photobiological solar and electrofuels, which are breaking innovative ground with the straightforward conversion of solar energy to biofuels, are also considered as fourth-generation biofuels. Resources for such biofuel production are cheap and available and are a product of the developmental progress of engineered crops through genetic engineering and the emerging field of synthetic biology [23].

This review paper presents an overview of very recent bioethanol production processes by enzymatic hydrolysis from different lignocellulosic biomass sources, such as marine algae, agricultural residues, and forest feedstocks.

## 2. Lignocellulosic Sources

Lignocellulosic sources are the world’s largest renewable sources for bioethanol production and can be divided into three main types: (1) marine algae, (2) agricultural residues and municipal solid wastes, (3) and forest woody feedstocks. Different groups of raw materials are available for bioethanol production, dependent on their structure and composition. Many research reports describe different lignocellulosic waste for the production of bioethanol, such as corn stover, rice straw, bagasse, grass, and others [8,24,25,26,27]. Marine algae are the new and promising alternative for the production of bioethanol, since they can grow fast, but they still face some challenges, such as their high pretreatment costs. The primary crop for bioethanol production is switch grass that grows in the northern hemisphere and is of great interest because of its low cost, as well as its abundance and high content of sugar substrates. Different grasses also require almost no or very low maintenance and no fertilization.

### 2.1. Marine Algae

**Figure 3:**
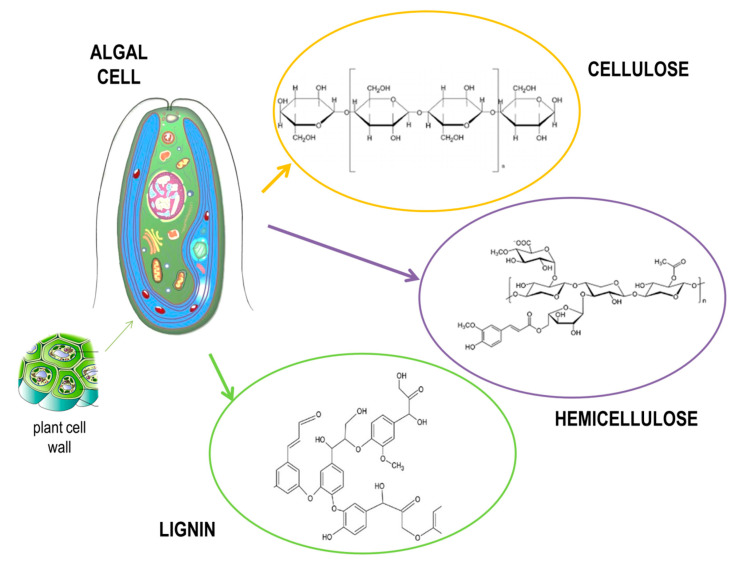
Structure of an algal cell.

Marine algae present a renewable biomass source, whose main advantage is in fast and sustainable growth [28,29]. Moreover, they are gaining interest as third-generation feedstock because of the rapid development of biorefineries designed for bioethanol production. Since marine algae have a high content of carbohydrates in their composition, they are able to yield almost 60 times more alcohol than other agricultural or forestry feedstocks [30]. However, there are still some challenges present in using algae as a source for bioethanol production, since the presence of hydrocolloid polymers present in the cell walls of algae makes the walls stronger and therefore requires a pretreatment of such algal feedstock to break down those complex cell wall structures (Figure 3). Such process is expensive and presents around 20% of production cost [31,32].

Macroalgae or seaweed are divided into three main types: brown algae (Phaeophyta), red algae (Rhodophyta), and green algae (Chlorophyta). In general, macroalgae contain around 25%–60% carbohydrates, 5%–20% proteins, 0.5%–5% lipids, and around 15%–40% ash. Regarding sugar composition (Table 1), brown algae generally consist of alginate, cellulose, mannitol, fucoidin, and laminarin, while red algae consist of agar, carrageenan, cellulose, and lignin; green alga consist of starch, cellulose, mannan, and ulvan [33,34]. In order to hydrolyze seaweed into fermentable sugars, pretreatment and saccharification processes are required. A typical pretreatment method for seaweed into hydrolysate conversion for bioethanol production is based on using acid pretreatment with fairly high temperatures (100–150 °C) [35]. However, other methods for the pretreatment of lignocellulosic bioethanol production, such as microwave [36] and alkali [37] pretreatments, were also used in hydrolysis processes. Enzymatic saccharification is often required following pretreatment using cellulosic enzyme solutions, such as alginate lyase or laminarinase, which are successful in the effective hydrolysis of brown algae [38,39]. On the other hand, microalgae have the ability to grow fast with high lipid content in some species, such as Chlorella sp. In addition, some species, such as Synechococcus sp., contain around 60% carbohydrates [40]. Factors influencing the lipid and carbohydrate content of microalgae are temperature, light, salinity, nutrient content, O_2_, CO_2_ level, and pH [41]. The microalgal cell wall is easily broken down with pretreatment with lysozymes or diluted acids when compared to macroalgae or other biomass. Cellulose, hemi-cellulose, lignin, pectin, and other carbohydrates converted to monomers by enzymatic or acid hydrolysis are the most common components in the microalgal cell wall [42].

Many microalgal strains can accumulate 30% of their dry biomass as lipids, while others can accumulate around 70% as carbohydrates, which are water-insoluble polysaccharides. Those make microalgae an attractive feedstock for different fermentation processes in biofuel production. Green algae can synthesize amylopectin-like polysaccharides in their chloroplasts, while the main polysaccharide is glycogen accumulated in cytosol. Cellulose is the main component of the cell wall; however, the cell wall lacks hemicellulose and lignin. Microalgae have many attractive advantages as feedstocks, such as higher biomass productivity and a noncompetitive nature with crops and other edible feedstocks, and they are far better at effective recycling and converting sunlight and CO2 to algal biomass [43].

### 2.2. Agricultural Residues and Municipal Wastes

There are four major agricultural wastes that are the most favorable biomass feedstocks for the production of bioethanol. Those are rice straw, wheat straw, corn straw, and sugarcane baggase, which are also used as animal fodder and domestic fuel [4]. Most potential feedstocks for bioethanol production are wheat and rice straws and corn stalks, since they contain approximately 35% hemicellulosic material [44]. As agricultural residues present an environmentally friendly step in the process, they also help prevent deforestation. Different crops go through short-term harvest rotations and are therefore more available for bioethanol production [4,45]. Rice straw disposal is limited by its slow degradation, big bulk material, and high mineral content. Only a small part of rice straw is used as animal feed, while the rest of rice straw (more than 90%) is removed by field burning. Among the four mentioned major agricultural wastes, rice straw can produce more than 200 billion liters biofuel per year, being the most abundant waste biomass feedstock in the world [46].

As an alternative to agricultural cellulosic residues, a good candidate for raw materials that has potential for bioethanol production is municipal solid wastes, which can solve the household garbage disposal and therefore limit the environmental problems that may occur due to such problems [47]. All kinds of high yielding crops are gaining great interest as an alternative to woody and agricultural residues, since they present almost 70% of total available feedstocks for bioethanol production.

### 2.3. Forest Feedstocks

Two types of forest feedstocks are available for bioethanol production: those are hardwoods and softwoods. Softwoods, such as pine, spruce, cypress, fir, and others have lower density and can grow on a higher rate, while hardwoods, such as oak, willow, poplar, cottonwood, and others are angiosperm and mostly deciduous [48]. Cottonwood is believed to be the most suitable woody feedstock for bioethanol production, since it is the most productive tree with several important advantages, such as a large amount of clones, restoration possibility by multiple cuttings, and uniformity in planting material quality [49]. Different forest feedstocks possess more lignin and less ash content, which makes such woody feedstocks a very attractive raw material to improve and increase bioethanol conversions in its production processes [50].

## 3. Lignocellulosic Biomass Composition

**Figure 4:**
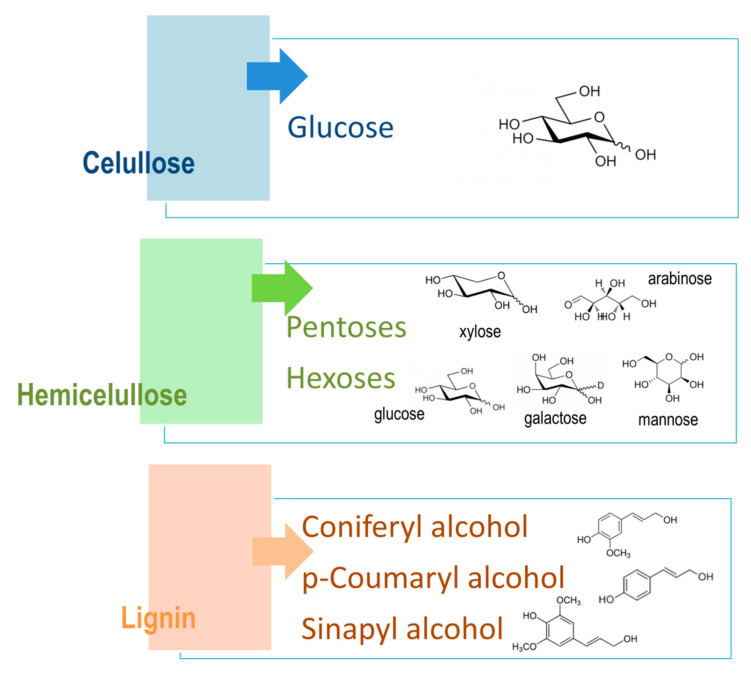
Cellulose, hemicellulose, lignin.

Lignocellulosic materials are divided into three main components: cellulose, hemicellulose, and lignin (Figure 4), where cellulose and hemicellulose together present around 70% of all biomass. Both cellulose and hemicellulose are closely connected to component lignin through covalent and hydrogen bonds, which make its structure more robust and treatment resistant [51].

### 3.1. Cellulose

**Figure 5:**
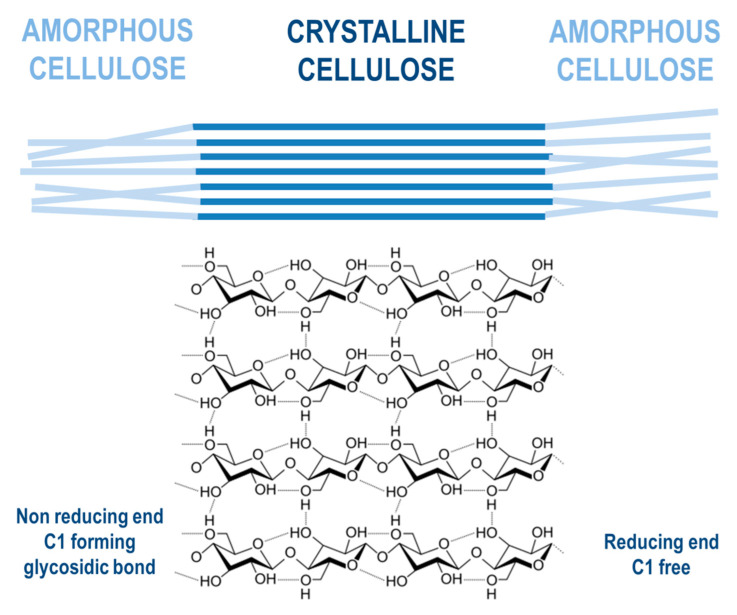
Cellulose structure.

Cellulose is a linear component that consists of long-chain glucose monomer β-D-glucopyranose linked with β-(1,4)-glycosidic bonds, which can also reach more than one thousand units of glucose in length, with cellobiose being its repeating unit. Cellulosic chains are composed of around 1000 D-glucose units, which are arranged in microfibrils. Those fibrils form a lignocellulosic matrix with hydrogen linkages, which makes it very resistant, strong, and compact in its structure [52]. Cellulose is organized by intermolecular and intramolecular hydrogen bonds, as well as van der Waals forces, which make the cellulose crystalline. Where weaker bonds occur in the structure, the cellulose structure is amorphous (Figure 5). Cellulose, being the most widespread organic polymer in nature, requires a temperature of around 300 °C to be converted to an amorphous structure from crystalline. There are two ending groups in each chain of cellulose: a non-reducing and a reducing group. The non-reducing group is at the closed ring structure, while the reducing group is present at the opposite end of the chain, consisting of an aliphatic structure and a carbonyl group [53,54]. Cellulose being highly abundant in plants can be synthesized by animals, algae, bacteria, and fungi as well [55]. A study by Sacui et al. describes the production of cellulose nanocrystals and cellulose nanofibrils by, among others, enzymatic hydrolysis from three types of raw materials: wood, tunicate, and bacteria [56]. A study by Uzyol et al. reports on the production of bacterial cellulose from algae *Chlorella vulgaris*, which was used as a source of glucose for the production of bacterial cellulose [57].

### 3.2. Hemicellulose

Hemicellulose is a heterogenous and amorphous structure of polymers that contains different monosaccharide subunits, which include d-glucose, d-mannose, d-galactose, d-xylose, and L-arabinose, as well as other sugar acids, such as d-galacturonic and D-glucuronic acids [58]. The structure of hemicellulose is amorphous and is not physically strong, being easily hydrolyzed by hemicellulose enzymes [59]. It was reported that hemicellulose removal in the pretreatment process can increase the cellulose conversion due to the accessibility of enzymes to cellulose [60]. Through aromatic esters, hemicelluloses can also be linked with lignin, as well as to celluloses with hydrogen bonds, therefore providing a bond between cellulose and lignin. Polymers of hemicelluloses are constituted of different sugars, which the hemicelluloses are classified after. For example, xylan consists of xylose units linked with β-1,4 bonds to the L-arabinose substituted unit of the xylopyranose unit [55,61].

### 3.3. Lignin

Lignin is an aromatic polymer that is linked with covalent bonds to different xylans. It is a very complex heteropolymer of phenylpropanoied units that is composed of phenolic monomers, such as coniferyl, coumaryl, and sinapyl alcohol. Lignin contributes to the rigidity of the structure and its hydrophobicity [62]. Lignin, the linking part between cellulose and hemicellulose in the cell walls, obstructs cellulose conversion because of several factors, such as total lignin content and lignin structure. Lignin acts as a physical barrier and can limit the accessibility to polysaccharides [63]. The highest levels of lignin are present in softwood, around 30–60%, while grasses and other agricultural wastes contain only around 10–30% of lignin. Components of lignin have a dilution effect when added together with solid components to the pretreatment process, and that affects the hydrolysis process. For that reason, lignin is gaining more and more interest in the hydrolysis process itself [64].

## 4. Pretreatment of Lignocellulosic Biomass

Biomass recalcitrance is defined as molecular, ultrastructural, and chemical recalcitrance, which limits the enzymatic efficiency and degradability of celluloses and hemicelluloses. Lignin, being the main recalcitrant, is the plant’s defense to prevent its degradation by hydrolytic enzymes. In order to overcome the recalcitrance of biomass, pretreatment is the main step in removing and isolating hemicellulosic and cellulosic polysaccharides, which can be used to produce biopolymers and biochemicals as well as biofuels. Nevertheless, lignocellulosic bioethanol production is characterized by following process points, such as pretreatment, enzymatic hydrolysis, and fermentation. There are different pretreatment methods available to convert plant polysaccharides into fermentable sugars. In order to obtain a successful and efficient pretreatment method, according to a study by Silveira et al., one must minimize the inhibitory compounds for enzymatic hydrolysis and fermentation, decrease the loading capacity of the enzyme in order to obtain efficient hydrolysis, avoid loss of sugar in pretreatment fractions, obtain lignin and other compounds’ recovery for ongoing conversion, and ensure the efficient use of energy. There are different pretreatment methods used for the disruption of plant cell walls, such as physical, chemical, physicochemical, and biological pretreatment methods. Physical pretreatment methods include ultrasound and milling, which reduces the particle size, crystallinity index, and polymerization degree. Chemical pretreatment methods involve acid pretreatments, which allow hydrolysis and the removal of hemicelluloses with the use of diluted acids. Alkaline pretreatments, with the use of alkaline solutions enable a high digestibility of cellulose while removing lignin or breaking bonds in the lignin carbohydrate complex. Organosolv pretreatment processes increase the volume of substrates’ pores and surface area. In addition, the use of new methods with nonaqueous and nonderivatizing solvents is possible, using green solvents, such as supercritical fluids and ionic liquids. Physicochemical pretreatment methods include ammonia fiber extraction (AFEX), hydrothermal processing, such as steam explosion or liquid hot water pretreatment, and pretreatment with sub- and supercritical fluids. Biological pretreatment methods use fungi, different microorganisms, and lignin-degrading enzymes, such as pectinases, lignin peroxidases, xylanases, mannanases, manganese peroxidases, and feruloyl esterases [65]. Pretreatments protocols can enhance cellulose access to enzymes during enzymatic hydrolysis, which consequently turns sugars into fermented ethanol. Therefore, enzymatic hydrolysis presents a more accessible path for cellulose and hemicellulose after pretreatment [66,67,68].

### Cellulases and Hemicellulases

Cellulases are o-glycoside hydrolases that can hydrolyze the β-1,4-d-glucan bonds of cellulose, where primary products are glucose, cellobiose, and cellooligosaccharides [69]. Cellulases have carbohydrate binding modules (CBM), which are responsible for the binding of enzyme–substrate, where the CBM is the non-catalytic protein attached to the catalytic domain. Cellulases that contribute to the degradation of cell walls of the plants are divided into 44 families. Endoglucanases (EG) initiate the enzymatic hydrolysis of celluloses, by which they reduce the degree of polymerization, as they split the chains in the amorphous regions of cellulose. Exoglucanases can produce cellobiose at reducing and non-reducing ends of cellulose chains, while β-glucosidases hydrolyze cellobiose to glucose molecules [70,71,72,73]. Hemicellulases are specific for the degradation of polysaccharides, which contain glucose, mannose, galactose, fructose, xylose, and different acids, such as galacturonic, acetic, ferulic, and others [74,75]. Hemicellulases are applicable in many different areas of biotechnology, such as in biofuel processes, where after the pretreatment step, the biomass is converted into bioethanol by processes such as fermentation and anaerobic digestion [76]. Both enzymatic hydrolysis and fermentation are taking place under the same conditions in a bioreactor, which is “simultaneous fermentation saccharification”. They can also be performed in separate steps as “saccharification and separate hydrolysis”, where the simultaneous hydrolysis of cellulose and hemicellulose is performed by the simultaneous saccharification and co-fermentation of two different monosaccharides pentoses and hexoses [77]. (Hemi)cellulases are used in many other technologies, such as in brewery, where they are used to reduce the viscosity of β-glucans and to increase the content of alcohol in beverages during the fermentation process of sugars. They can also be used in the production of various other drinks, such as wines, juices [78], and in the production of olive oil [79].

## 5. Enzymatic Hydrolysis

**Figure 6:**
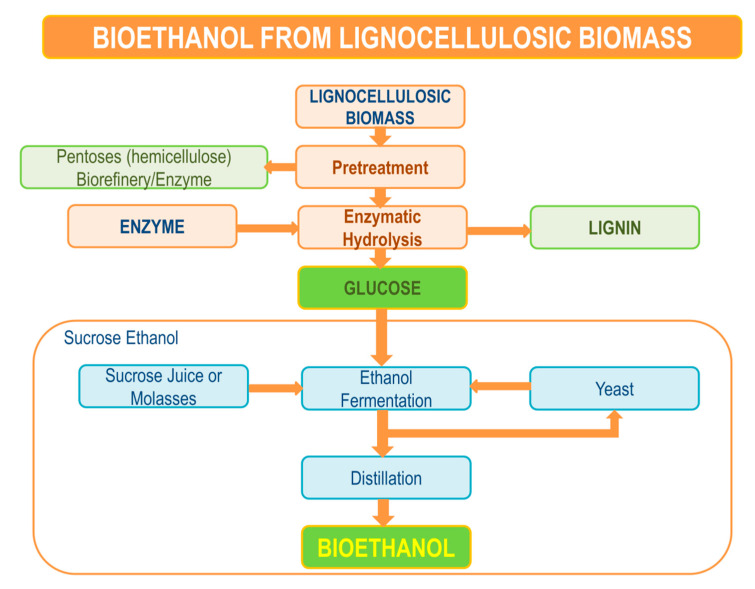
Enzymatic hydrolysis pathway.

Enzymatic hydrolysis presents an important process in the conversion of cellulose in pretreated biomass. Its pathway is presented in Figure 6. The conversion of cellulose to glucose is performed by cellulase enzymes under mild conditions, such as temperature from 40 to 50 °C and pH around 4.5 and 5. An important role in the efficiency of hydrolysis presents the pretreatment process of lignocellulosic biomass. Such pretreatment process includes lignin removal, hemicellulose solubility process, duration of hydrolysis, and enzyme loading. The crystalline structure of cellulose affects the rate of hydrolysis, since the hemicellulose and its present lignin make a bond with cellulose and therefore limit the process of hydrolysis. Non-ionic surfactants or polymers containing poly(ethylene glycol) such as PEG are known to enhance the efficiency of hydrolysis, since they can change the surface properties of cellulose and therefore lower the enzyme loading [11]. As per study by Börjesson, adsorbed PEG on lignocellulose is due to hydrophobic PEG and the hydrogen part of lignin in lignocellulose, where PEG, as other poly(ethylene oxides), adsorbs to lignin. Such adsorbed polymers of PEG on the surface of lignocellulose, which result in excluded volume interactions, can obstruct enzymes binding to lignin surface. Therefore, high enzyme concentrations are available for the degradation of cellulose [13]. Furthermore, the addition of PEG was proven to facilitate enzymatic hydrolysis of lignocellulosic materials in a study by Li as well, where PEG positively affected the hydrolytic enzyme activity of materials of pure cellulose without lignin [80]. Sugar yields in the enzymatic hydrolysis process increased with the addition of PEG in a study by Cheng as well [81]. PEG also facilitated the highest enzymatic hydrolysis yield in a study by Zambare [82]. Another aspect of improving the bioethanol production through enzymatic hydrolysis is to improve the operability of hydrolysis by higher substrate concentrations, which affects the rate of hydrolysis in order to maximize glucose yields in hydrolysate [83,84,85]. A study by Ostadjoo et al. reports of innovative enzymatic protocol using xylanase from *Thermomyces lanuginosus*, which enables hemicellulose hydrolysis, where different substrates of different concentrations were used, such as xylans from oat spelt and birchwood, wheat straw biomass, and sugarcane bagasse [84]. Wang et al. investigated a two-step pretreating protocol, where NaOH and ozone were used on corn stover in order to improve enzymatic hydrolysis [85]. Moreover, efficient conversion of the carbohydrates to bioethanol can be reached with the optimization of process parameters, such as solid loading, enzyme loading, as well as shaking speed and hydrolyzation time.

The fermentation process can be achieved over continuous, batch, and fed-batch fermentation, while fed-batch in stirred tank is the primary choice in industrial fermentations because of its ability to provide optimal conditions. Glucose fermentation with the use of robust industrial host strains can elevate yields of ethanol due to its high specific ethanol productivity. Such strains are *Zymomonas mobilis* and *Saccharomyces cerevisiae*., although such fermenters can not use pentoses. Traditionally, pentose fermenting yeasts are *Candida shehatae, Scheffersomyces stipitisa,* and *Pachysolen tannophilus* [86,87]. However, some drawbacks are present when using these microorganisms with xylose fermentation, which are described in a study by Wirawan et al. [88].

### 5.1. Factors Influencing Enzymatic Hydrolysis

#### 5.1.1. Crystallinity of Cellulose

Crystallinity is the most studied property of cellulose and presents the ratio between crystalline and amorphous regions of cellulose. Crystalline fibers in cellulose are linked with non-covalent hydrogen bonds, which make the enzymatic hydrolysis process much easier and fluent compared to amorphous regions of cellulose. Different studies show the different impact of crystallinity on the hydrolysis process. Alkaline pretreatment is able to solubilize the amorphous hemicellulose and lignin that is linked with cellulose by destroying ester linkages, which are exposed to enzymes. Ling et al. explored the crystal structures and crystallinity of alkaline pretreated cellulose, following enzymatic hydrolysis, where such a process resulted in an increased crystallinity of cellulose. Alkaline pretreatment (or mercerization completion) occurred under high alkaline concentration of 14-18% of NaOH, which caused the deformation of crystalline cellulose. The study also provided valuable information for the enzymatic digestibility of studied lignocellulosic materials [89]. Alkaline treatment is also reported to minimize the degree of polymerization, which consequently disrupts the crystalline structure of cellulose, therefore promoting the accessibility and digestibility of enzymatic hydrolysis, as studied by Wada et al., where the efficient conversion of cellulose was demonstrated by a simple mercerization process [90].

#### 5.1.2. Particle Size of Lignocellulosic Biomass

The particle size of lignocellulosic biomass plays an important role in the efficiency of the hydrolysis process, which affects its reaction rate and mass transfer, as well as the fermentation process. Structural changes of lignocellulosic biomass can be done with cutting, grinding, milling, and extrusion, which enhance the enzyme–cellulose affinity and therefore increases the rate of hydrolysis [91]. Particle size affects the kinetics diffusion and the pretreatment efficiency, sugar yield, as well as the removal of lignin. While smaller particles have a larger surface area, they can still be difficult to handle. On the other hand, larger particles may not undergo complete pretreatment interiorly, which can cause problems in the hydrolysis process. Therefore, it is essential to optimize the particle size of biomass in order to achieve high conversions with low cost production. The effects of rice straw particles on pretreatment efficiency with diluted acid, as well as on enzymatic hydrolysis, were studied by Kapoor et al. The particle size affected glucan hydrolysis and sugar recovery [92]. A study by Li et al. reports on how the particle sizes of sludge decreased by more than 60% as a result of a thermal hydrolysis pretreatment, therefore increasing the amount of sulfur components and also proving how free ammonia nitrogen and thermal hydrolysis pretreatment affect the conversion of volatile sulfur compounds. While thermal hydrolysis pretreatment had no effect on the involving enzymes in conversion, it did reduce the particle sizes of sludge [93]. A study by Wang et al. reveals a change in particle size distribution as a result of twin-screw extrusion pretreatment, while having no effect on the chemical structure of corn stover samples. With such a method, the crystallinity index of corn stover samples was decreased as well [94]. Another study by Lan et al. shows the effect on particle size, crystallinity, electrostatic charge, hydrogen bonds, and hydrophobicity while studying the efficiency of enzymatic hydrolysis of sugarcane bagasse treated with p-toluenesulfonic acid and the amount of cellulose adsorption of Avicel [95].

#### 5.1.3. Accessible Surface Area and Pore Volume of Lignocellulosic Biomass

In relation to the porosity structure of lignocellulosic biomass, accessible surface area presents an important factor that influences the process of enzymatic hydrolysis. In addition, pore volume or pore size influences enzymatic hydrolysis, since the increase in pore volume increases the accessible surface area as well. Therefore, the pretreatment of different lignocellulosic biomass highly influences enzymatic conversion with increased accessible surface area. Accessible surface area is hard to determine, but specific surface area is often an indicator used to measure how much surface is available to the enzymes. However, higher specific surface area is a consequence of smaller particle sizes of lignocellulosic biomass [67,96,97,98]. Depending on the size and shape of pores, the accessible volume or pore size of cellulose is influencing the enzymatic hydrolysis of lignocellulosic biomass, and the volume of the pore is more or less accessible to the enzyme as well [99,100,101]. To make lignocellulosic biomass more digestible by enzymes, pretreatment with ionic liquids chemical alterations can affect enzymatic digestibility. A study by Torr et al. shows glucan conversions from 20 to 80% on substrates saccharification [96]. A study by Lu et al. evaluated the physicochemical properties effect of ball-milled cellulose on cellulase adsorption and glucose yield, where it affected specific surface area, particle size, crystallinity, and polymerization degree. With prolonged ball-milling, cellulose had decreased enzyme adsorption capacity and increased initial hydrolysis rate [98]. Peciulyte et al. investigated how the average pore sizes of the starting material affect enzymatic conversion yields. In addition, crystallinity degree was obtained during the enzymatic hydrolysis of substrates with high cellulose content. Bigger pore sizes in substrates provided higher conversion yields, as reported [101].

### 5.2. Enzymatic Hydrolysis Pathway

#### 5.2.1. From Marine Algae

Since marine algae are attracting more and more interest as alternative feedstocks for bioethanol production, due to their ability of fast growth, there are many research articles where enzymatic hydrolysis is used to produce third-generation biofuels. A study by Shokrkar et al. developed a kinetic model of enzymatic hydrolysis using microalgal cellulose, where two reactions were carried out, starting with the hydrolyzation of algal cellulose to cellobiose and glucose, and later on, a breakdown reaction of cellobiose to glucose. They obtained 57% of glucose yield at 50 g/L of microalgal biomass using *Chlorococcum* sp, 50 °C and pH of 5. Enzyme cellulase was applied three times without affecting the glucose yield. In addition, the microalgal glucose yielded 0.46 g/g glucose when converted into ethanol [102]. Onay demonstrated using *Hindakia tetrachotoma ME03* as microalgal biomass to produce bioethanol, where enzymatic hydrolysis was researched as well. Enzymatic hydrolysis was performed using enzymes β-glucosidase/cellulase and α-amylase with a saccharification yield of 92%, which demonstrated that *H. tetrachotoma* ME03 is a promising candidate for the production of bioethanol [103]. Another study by Ngamsirisomsakul et al. reports on *Chlorella* sp. as possible microalgal feedstock for bioethanol production, which was investigated with enzymatic hydrolysis using enzymes α-amylase and glucoamylase, where *Chlorella* sp. was able to produce up to 11 g/L of bioethanol [104]. A study by Kumar et al. investigated macroalgal biomass from fresh river water, where enzymatic hydrolysis was performed with cellulose enzyme and the bioethanol yielded 61% [105]. Another study used *Spirulina platensis* algae fir biomass in the saccharification and fermentation process for bioethanol production. The enzymatic hydrolysis of algae polysaccharides yielded above 80%, which proves that different algae are promising alternatives to renewable energy contributions [106]. Using fungi in the pretreatment of marine algae can increase the ethanol yield in enzymatic hydrolysis process up to 38%, where a study managed to increase sugar yield 2.3-fold [107]. Macroalgae, such as seaweed *Ulva* sp. (*Chlorophyta*), was used to optimize the enzymatic hydrolysis of dried mass by enzymes cellulase, amyloglucosidase, and α-amylase, which yielded in 77% of ethanol [108]. All studies are summarized in Table 2.

#### 5.2.2. From Agricultural Wastes and Residues

When investigating bioethanol pathways through enzymatic hydrolysis from different wastes and residues, many different agricultural wastes have been used. Hornbeam residues were investigated using commercial enzyme cellulose (Cellic Ctec2), where glucose yield was investigated by varying three parameters, such as severity factor of pretreatment, total solids of enzymatic hydrolysis, and enzyme loading. The optimization of those parameters resulted in 68% sugar yield, which corresponds to ethanol production of around 250 L/ton of dry raw material [109]. Black tea waste was also investigated, where two different yeasts (*Zygosaccharomyces bailii* MTCC 8177 and *Brettanomyces claussenii* MTCC 7801) were used for bioethanol production. Enzymatic hydrolysis was performed using an enzyme cocktail from Novozyme, containing different carbohydrases. *Z. bailii* was reported to be a better medium, since it obtained 6-fold greater glucose yield than from the *B. claussenii* [110]. Sisal waste was investigated for bioethanol production, where enzymatic hydrolysis was performed using cellulase C1794, which resulted in 92% of glucose conversion [111]. Pomegranate peels were used as a substrate for bioethanol production, using cellulase enzyme, where glucose conversion resulted in 95% yield [112]. In addition, banana waste was used as a substrate, where enzymatic hydrolysis was optimized to obtain around 100 g/L concentration of glucose, using enzyme glucan. The ethanol yield resulted in 87% [113]. Gooseweed (*Sphenoclea zeylanica Gaertn*.) was investigated as a potential substrate for bioethanol production, where enzymatic hydrolysis was performed using enzyme β-glucosidase, where the highest bioethanol concentration resulted in 11.84 g/L after five days [114]. Corncob is another substrate used with enzymatic hydrolysis for bioethanol production, where thermostable endo-xylanase was used. After 96 h of enzymatic hydrolysis, hydrolysate resulted in 62 g/L of total sugar, 51 g/L of glucose, 10 g/L of xylose, and 0.9 g/L of arabinose [115]. Another suitable substrate for bioethanol production is potato peel waste, where commercial enzymes cellulase and amylase were used for enzymatic hydrolysis treatment. Bioethanol yield using commercial enzymes resulted in 96% [116]. Improved enzymatic hydrolysis was performed using *Agave salmiana* leaves, which were pretreated with acid-alkaline process before the process of enzymatic saccharification. Enzymatic hydrolysis was performed using commercial enzyme Celluclast, containing cellulase and β-glucosidase. Conversion of 95% was reached by enzymatic treatment, releasing sugar concentrations of 50 g/L [117]. Vetiver grass that is rich lignocellulosic material was used with celluloytic enzymes, such as carboxymethyl cellulose and β-glucosidase for bioethanol production. The highest sugar content from enzymatic hydrolysis and bioethanol production was obtained with 21 g/L and around 6 g/L, respectively [118]. Corn straw was investigated as a raw material in simultaneous saccharification and fermentation, which was performed as the simultaneous enzymatic hydrolysis of cellulose and obtained sugars, where a complex of enyzmes was used, such as cellulase, arabanase, b-glucanase, hemicellulose, and xylanase. The process resulted in 17 g/L of ethanol, which yielded 31% [119]. To improve ethanol production, pomegranate peels were used as substrate for enzymatic hydrolysis, in which process different cellulase concentrations were used. The study increased ethanol production to 13 g/L, where 98% ethanol yield was reached, which improved fermentation efficiency [120]. In addition, fungal enzymes were used in the enzymatic hydrolysis process of cellulose to sugars, which can be fermented to ethanol. Raw material used was barley straw, and the study concluded that fungi can also be a valuable source for a cost-effective production of enzymes that can be applied in enzymatic hydrolysis process for an efficient bioethanol production [121].

#### 5.2.3. From Wood Feedstocks

As a renewal energy source for bioethanol production, different wood feedstocks offer major benefits as possible substrates for bioethanol production. Table 3 shows a comparative chart for bioethanol production from different wood feedstocks. Palm wood was used as substrate in enzymatic hydrolysis, using enzyme cellulose, where experimental bioethanol yielded 23 g/L at given conditions [122]. Various hardwoods were pretreated with hydrogen peroxide, which highly improved hydrolysis efficiency due to reducing lignin content. Enzymatic hydrolysis was performed using glucan cellulase, which contributed to the increase in production of bioethanol [123]. In addition, poplar wood was used as substrate and glucan cellulase was used in enzymatic hydrolysis, where 68% yield of fermentation efficiency suggests that acid hydrotrope fractionation has potential in forestry feedstocks for bioethanol producton [124]. Willow biomass was pretreated with steam explosion process, after enzymatic hydrolysis was performed using cellulase enzyme, which resulted in a cellulose to glucose conversion of 80% [125]. At low temperatures, pretreated sugarcane bagasse was enzymatically hydrolyzed, using glucan enzyme, which yielded 74% of glucan conversion, which also suggests promising results for bioethanol production [126]. Enzymatic hydrolysis was optimized based on sawdust from Ayous tree, using enzyme cellulase. After enzymatic hydrolysis, a decrease in substrate loading occurred, which led to an efficient hydrolysis yield of cellulose. The conversion yield resulted in 69% [127]. Another study investigated sawdust for bioethanol production as well, originating from softwood. The study investigated the enzymatic hydrolysis of pretreated sawdust using commercial enzyme Cellic Ctec2, which followed fermentation. The hydrolysis achieved 80% saccharification yield and a 80% glucose to ethanol conversion yield [128].

Many different biomass sources have been used for bioethanol production. However, the final yield of ethanol from certain lignocellulosic biomass is highly dependent on conversion efficiency, which also depends on the process conditions, nature of used biomass, and fermentation process with various microorganisms. Additionally, the bioethanol production from different lignocellulosic materials is also determined by glucan hydrolysis efficiency. For example, among agricultural wastes and residues, bagasse has the highest glucan digestibility (47%), followed by corn stover (23%), wheat straw (11%), and rice straw (10%). In comparison, different crops have much lower glucan digestability, such as switchgrass (17%) and bamboo (3%).

From the presented research articles, it can be seen that various marine algae are being used as feedstocks to produce bioethanol in the most efficient way possible. Among mixed micro- and macroalgae feedstocls, also *H. tetrachotoma ME03, Chlorella sp., Spirulina platensis, K. alverzii, G. amansii,* and *Ulva sp*. are being utilized and extensively investigated as potential feedstocks for bioethanol production. Such micro- and macroalgal biomass is being pretreated with many innovative processes, such as fungal or hydrothermal pretreatment and acidic, alkaline, or enzymatic hydrolysis, using various native or commercial enzymes. Among agricultural residues and wastes, many were utilized, from hornbeam residue, black tea, and sisal waste to pomegranate, potato, and banana peels. Many wood feedstocks were utilized as well, such as palm and poplar wood, sawdust and sawmill mixed feedstocks and others, pretreated with hydrogen peroxide acetic acid, acid hydrotrope, steam explosion, aqueous ammonia soaking, and the Organosolv process.

## 6. Fermentation

The pretreatment process as well as the hydrolysis process are of key importance to an optimized fermentation process [129]. Since fermentation is a natural pathway, it still requires microorganisms to convert fermentable sugars into alcohol (or lactic acid or many other different end products). For such purposes, many industrial yeasts, such as *S. cerevisiae* were used, mostly in wine, brewery, and other alcohol-producing industries [130]. *S. cerevisiae* as the main fermentative strain is also used in sugar-based biofuel industries. The cellulosic material or slurry, when pretreated, is converted to fermentable sugars, once becoming available for either enzymatic or acidic hydrolysis. When such slurry is mixed with water, it forms a broth. Usually in such batch fermentation, *S. cerevieiase* is used to ferment hexose sugars, such as glucose, into ethanol under anaerobic conditions and at controlled temperature. During such yeast fermentation, CO_2_ by-products are formed, which are supplemented by nitrogen to accelerate the reaction. In such fermentation processes, high ethanol yields can be achieved by *S. cerevisiae* from hexose sugars. However, although a wide tolerance is characteristic for *S. cerevisiae*, it is not able to ferment other sugars than glucose [131,132]. One similar microorganism is also *Zymomonas mobilis*. A lot of lignocellulosic materials contain mostly pentose sugars, such as D-xylose, which *S. cerevisiae* and *Z. mobilis* can not ferment. Natural xylose fermenting yeasts (such as *Pichia stipites, Candida parapsilosis*) can metabolize xylose through xylose reductase action, where xylose is converted to xylitol and through xylitol dehydrogenase, where xylitol is converted to xylulose [27]. Moreover, a microorganism with optimal fermentative characteristics should endure high alcohol content and chemical inhibitors that are formed in pretreatment and hydrolysis process. Therefore, many genetically engineered microorganisms were developed, which can ferment pentose and hexose sugars simultaneously [133]. In addition, thermophilic anaerobic bacteria were investigated for bioethanol production, such as *Thermoanaerobacter ethanolucus, Thermoanaerobacter mathranii, Thermoanaerobacter brockii*, and others. Such bacteria are able to overcome extreme temperatures, while pretreating inexpensive biomass feedstocks. However, the main disadvantage of such bacteria is their low ethanol tolerance [134].

The fermentation process can be performed as a continuous, batch, or fed-batch process, which depends on lignocellulosic hydrolysate and on the kinetic properties of microorganisms used. In the continuous process, substrate containing feed and culture medium are continuously pumped into reactor, which holds active microorganisms. The batch process is a simple method and considered a closed culture system that contains an initial nutrient amount that is limited. Such culture is inoculated with microorganisms that perform fermentation. Fed-batch reactors are frequently used in industrial applications due to many advantages from both continuous and batch processes. The fed-batch process can increase the concentration of maximum viable cells, can extend the lifetime of culture used, and can accumulate product with higher concentration [135].

There are different techniques and strategies used to combine hydrolysis and the fermentation step in one single reactor [136,137]. Separate hydrolysis and fermentation (SHF), where enzymatic hydrolysis is separated from the fermentation step has the ability to perform each step under optimal conditions [138,139]. Simultaneous saccharification and fermentation (SSF) is an effective strategy when producing bioethanol from lignocellulosic materias, where diluted acid is combined with high temperatures in pretreatment processes. SSF process enhances the hydrolysis rate as well as decreases enzyme loading, which results in increased bioethanol yields with no or little contamination risk. The main advantages of the SSF process are increasing hydrolysis rates by the conversion of sugars, low enzyme content with higher yields of product, as well as shorter process duration with smaller reactor volumes. In addition, the microorganisms consume sugars immediately, which results in low sugar concentrations in fermenters [140,141]. The direct microbial conversion (DMC) process incorporates the production of cellulose, hydrolysis of cellulose, and fermentation of glucose into one single step. Such a process simplifies the production process and reduces the number of reactors, which leads also to reduced costs [5].

## 7. Conclusions and Future Perspectives

Currently, the enzymatic hydrolysis process is still a narrow way to the efficient production of bioethanol because of the high cost of many enzymes as well as the inhibitory properties of compounds that reduce the efficiency of the glucose production. Further research and new protocols are required to increase cellulose to glucose conversions by finding suitable lignocellulosic biomass structures that can improve bioethanol production. However, the main obstacle is the complex structure of lignocellulosic materials, which are the crystallinity of cellulose and issues that are related to lignin. All those properties make the enzymatic hydrolysis a challenging process. Therefore, suitable pretreatment protocols must be developed that can increase the efficiency of enzymatic activity, which can improve involving substrates for cellulolytic enzymes. Limitations are present in all steps of the process: pretreatment, enzymatic hydrolysis, fermentation, and saccharification processes need considerable new research strategies to improve the economics and efficiency of the process. Enzymatic hydrolysis is a cost-efficient process that increases the value of new by-products, derived from conversion, that are microbiologically-wise safe ingredients in food or products, as well as have increased nutritional and functional value. In addition, enzymatic hydrolysis still has many possibilities for improving enzyme production, its recycling, as well as genetic screening. While current biomass utilization has lignin being used for powering process energy necessities, it also gives lignin new possibilities in the industry of biorefineries. On the other hand, replacing different substrates with lignocellulosic and algal biomass is a step forward in using renewable sources, which reduce the current demands for food crops. Moving to the fourth generation of bioethanol production using cultivated algae will provide improvements that will benefit both environment and the production industries, which can promote more economical strategies for bioethanol production. However, there are numerous variables that affect the efficiency of conversion, such as source of biomass used for the production of a biofuel, pretreatment method, source of the enzyme, and its mixture used in the enzymatic hydrolysis. All of these features must be taken into account when designing the lignocellulosic conversion process to optimize its conditions.

## Figures and Tables

**Table 1:** Different macroalgae sugar composition.

Macroalgae	Sugar Composition
Phaeophyta (brown)	alginate cellulose mannitol fucoidin laminarin
Rhodophyta (red)	agar carrageenan cellulose lignin
Chlorophyta (green)	starch cellulose mannan ulvan

**Table 2:** Comparative chart for bioethanol production from marine algae.

Microalgal Biomass	Pretreatment	Enzyme	Yield	Ref.
Mixed microalgae	enzymatic hydrolysis	cellulase from *T. reesei*	57% glucose	[102]
*Hindakia tetrachotoma ME03*	acidic, alkaline, enzymatic hydrolysis	β-glucosidase from *E. coli*, cellulase from *A. niger*, α-amylase from *B. licheniformis*, amyloglucosidase from *A. niger*	92% sacchar.	[103]
*Chlorella* sp.	hydrothermal pretreatment	α-amylase, glucoamylase	11 g/L of bioethanol	[104]
Microalgal biomass	acid hydrolysis	cellulase from *T. reesei*	61% bioethanol	[105]
*Spirulina platensis*	none	α-amylase, amyloglucosidase	80% polysacchar.	[106]
*K. alvarezii,* *G. amansii*	fungal	Cellic CTec2	38% ethanol	[107]
*Ulva fasciata, Ulva rigida, Ulva ohnoi*	none	cellulase, amyloglucosidase, α-amylase	77% ethanol	[108]

**Table 3:** Comparative chart for bioethanol production from wood feedstocks.

Biomass	Pretreatment	Enzyme	Yield	Ref.
Palm wood	hydrothermal technique in conjunction with chemical method for removal of lignin	cellulase from *T. reesei*	23g/L bioethanol yield	[122]
Hardwood: **fringe** (*Chionanthus retusus*) **zelkova** (*Zelkova serrata*), **maple** (*Acer palmatum*) **chestnut** (*Castanea crenata*) **false acacia** (*Robinia pseudoacacia*)	hydrogen peroxide acetic acid pretreatment	cellulase (celluclast)	81% ethanol yield	[123]
Poplar wood	acid hydrotrope	CTec3 cellulase	68% bioethanol yield	[124]
Willow (*Salix viminalis* W)	steam explosion	cellulase from *T. reesei*	65% ethanol yield	[125]
Sugacane bagasse	low temperature aqueous ammonia soaking	Cellic CTec2 cellulase	91% ethanol yield	[126]
Sawdust from Ayous (*Triplochiton scleroxylon*)	Organosolv process	cellulase	69% enzymatic hydrolysis yield	[127]
Sawmill mixed feedstock	microwave-assisted water/ethanol Organosolv pretreatment	Cellic CTec2 cellulase	80% ethanol yield	[128]
